# Impact of Methods of Preparation on Mechanical Properties, Dissolution Behavior, and Tableting Characteristics of Ibuprofen-Loaded Amorphous Solid Dispersions

**DOI:** 10.1155/2024/2303942

**Published:** 2024-05-28

**Authors:** Ajam Uddin, Shimul Halder, Nandita Deb, Harinarayan Das, Madhabi Lata Shuma, Ikramul Hasan, Manik Chandra Shill, Syed Shabbir Haider

**Affiliations:** ^1^Department of Pharmaceutical Technology, Faculty of Pharmacy, University of Dhaka, Dhaka 1000, Bangladesh; ^2^Department of Physics, Faculty of Science, University of Dhaka, Dhaka 1000, Bangladesh; ^3^Materials Science Division, Atomic Energy Centre, Dhaka, Bangladesh; ^4^Department of Pharmacy, School of Pharmacy and Public Health, Independent University, Dhaka 1229, Bangladesh; ^5^Department of Pharmaceutical Sciences, North South University, Dhaka 1229, Bangladesh

## Abstract

This study aims to improve the biopharmaceutical, mechanical, and tableting properties of a poorly soluble drug, ibuprofen (IBP), by preparing amorphous solid dispersion (ASD) followed by a sustained-release tablet formulation. A suitable polymer to develop an ASD system was chosen by utilizing the apparent solubility of IBP in various polymer solutions. ASDs containing various ratios of IBP and selected polymer were prepared by the melt fusion (MF) method. ASD containing optimized drug-polymer ratio prepared by freeze-drying (FD) method was characterized and compared physicochemically. The solubility of IBP in water increased 28-fold and 35-fold when formulated as ASD by MF and FD, respectively. Precise formulations showed amorphization of IBP and increased surface area, improving solubility. The dissolution pattern of optimized ASD-IBP in pH 6.8 phosphate buffer after 60 min in MF and FD was enhanced 3-fold. In addition, direct compression tablets comprising optimized ASD granules from MF and FD were made and assessed using compendial and noncompendial methods. ASD-IBP/MF and ASD-IBP/FD formulations showed a similar drug release profile. In addition, 12 h of sustained IBP release from the ASD-IBP-containing tablets was obtained in a phosphate buffer with a pH of 6.8. From the dissolution kinetics analysis, the Weibull model fitted well. The drug release pattern indicated minimal variations between tablets formed using ASD-IBP prepared by both procedures; however, pre- and postcompression assessment parameters differed. From these findings, the application of ASD and sustained-release polymers in matrix formation might be beneficial in improving the solubility and absorption of poorly soluble drugs such as IBP.

## 1. Introduction

Ibuprofen (IBP), a classical nonsteroidal anti-inflammatory drug, is widely used to treat mild to moderate pain and fever. Due to its poor solubility, IBP is categorized as a BCS class-II drug [1, 2] with a pH dependency in the solubility profile [3]. In addition, IBP has a short elimination half-life of approximately 1.8–2 h [4]. Thus, poor solubility and quick elimination half-life limit in its clinical application, and a multiple-dosage regimen is required to maintain optimum plasma drug concentration. Approximately 25–40% of the current drugs and recently developed active pharmaceutical ingredients (APIs) exhibit limited solubility in water [5]. Consequently, it is necessary to administer a substantial dose of the medication to achieve a therapeutically significant plasma concentration of drugs with low solubility. Inadequately soluble drugs administered in substantial amounts have reduced systemic bioavailability and heightened local toxicity at sites of aggregate deposition owing to their elevated concentration [6]. Such problems may be resolved with a formulation technology that increases the solubility of the drug [7]. Several approaches can be employed to enhance the solubility of poorly soluble drugs in water. These include using surfactants, pH adjustment, nanosuspension technology, hydrotrophy, solid dispersion, and salt formation [8]. The solid dispersion (SD) approach is commonly employed to enhance the aqueous solubility, dissolution rate, and consequently, the bioavailability of drugs exhibiting limited solubility, among the previously mentioned methods [9].

Generally, SDs involve the incorporation of the drug into a pharmacologically inert hydrophilic carrier. The physical state of the drugs in SDs can be crystalline or amorphous. When the drug is amorphous, it can be dispersed over the carrier employed at a molecular level or incorporated in the solid dispersion as particles [10, 11]. On the other hand, when the drug is in its crystalline state, it can be solely incorporated as a particle. Theoretically, the drug and the carrier could form mixed crystals [12]. According to various studies, mixed crystals of this kind have never been encountered with SDs. By incorporating the molecular dispersion of one or more APIs in an inert carrier, SD increases the drug's solubility [13]. The chemical and physical stability of SDs, as well as their solubility, dissolution, bioavailability, and manufacturability, are all critical to the development of pharmaceuticals. [14]. Therefore, the mechanical properties of SDs may impact the compaction properties of the final dosage form formulation and the success of tablet manufacturing. From this perspective, knowledge of the mechanical properties of SDs is crucial for their rapid development into good-quality tablets [15]. Along with evaluations of stability and dissolution performance, mechanical property characterization of SD and its associated formulation blend should ideally be a key component of SD formulation design and optimization. However, very little is known about the SD system's potential to improve the biopharmaceutical and mechanical behavior of IBP.

Sustained-release (SR) systems have long been used to keep therapeutically effective plasma drug concentrations of the drug with a short biological half-life over a long period [16]. Therefore, SR dosage forms containing SD granules are an appealing formulation strategy for increasing the dissolution rate of poorly soluble drugs having a short biological half-life [17].

The primary objective of this research is to develop IBP-loaded ASD (ASD-IBP) to enhance the physicochemical properties and dissolution behavior of IBP. Furthermore, a sustained-release matrix tablet formulation containing the precised ASD granules was prepared and evaluated physicochemically.

## 2. Materials and Methods

### 2.1. Materials

BASF, Dhaka, Bangladesh, kindly donated working samples of IBP, Soluplus®, Kolliphor® P188, and Kolliphor® P407. Moreover, StarTab® (directly compressible starch) was a generous gift from Colorcon Bangladesh. All other chemicals (Eudragit® RSPO, Kollidon® SR, Aerosil® 200, Talc, magnesium stearate, di-sodium hydrogen phosphate, potassium dihydrogen phosphate, and sodium hydroxide pellets) were purchased commercially and were of pharmaceutical grade. The solvents (methanol, tert-butyl alcohol) used were of analytical grade.

### 2.2. IBP Content Determination

A 1 : 1 ratio of methanol to phosphate buffer (pH 6.8) solution was used to dissolve 10 mg of IBP in an adequate quantity of solvent to prepare a stock solution of IBP. The resulting solution had a final concentration of 100 *µ*g/mL. The aforementioned solutions were generated by serial dilution techniques to provide working solutions with concentrations ranging from 4 to 24 *μ*g/mL in methanol and 4 to 20 *μ*g/mL in phosphate buffer. The absorbance of the working solutions was measured using a UV-visible spectrophotometer (EMC-61PC-UV, EMCLAB, Germany) at a wavelength of 221 nm [18]. The average absorbance value of three runs for each concentration was plotted against respective drug concentrations, and accordingly, a standard curve was generated. The mean regression equation was *y* = 0.0455*x* + 0.2914 (*R*^2^ = 0.9962; *p* < 0.0001). The 95% CI estimated for the intercept value was 0.2558 to 0.3271. The equation derived from the standard curve was used to calculate the amount of IBP.

### 2.3. Phase Solubility Study

The apparent solubility of IBP in polymer solutions was assessed in triplicate using a well-established method described by Higuchi and Connors [19] to choose an appropriate carrier. At a polymer concentration ranging from 5 to 25 mg/mL, an estimated quantity of 20 mg of IBP was added into 10 mL of aqueous solutions containing Soluplus®, Kolliphor® P188, and Kolliphor® P407, respectively. The tubes were sealed and subjected to agitation at 75 rpm per minute for 48 h at 37°C using a shaking water bath (WBS-C1 Water Bath Shaker, China). Following a 48 h period, the samples were subjected to a settling duration of 10 minutes before undergoing centrifugation with a force of 10, 000 × *g* for 5 min. The supernatants obtained were subjected to filtration, and the content of IBP was analyzed using the procedure outlined in the preceding section.

The stability constant (*K*_*s*_) and complexation efficiency (C.E.) were determined by employing the given equations [20]:

(1) $$\begin{array}{c} K_{s}=\left(\frac{\text{slope}}{S_{0}\left(1-\text{slope}\right)}\right),\\ C.E.=\left(\frac{\text{slope}}{\left(1-\text{slope}\right)}\right). \end{array}$$

IBP's equilibrium aqueous solubility, or *S*_0_ can be found by graphing the concentration of IBP against various polymer concentrations.

### 2.4. Preparation of ASD-IBP

The amorphous solid dispersion of IBP (i.e., ASD-IBP: ASD1, ASD2, ASD3, and ASD4) with different ratios of carrier (2 : 1, 1 : 1, 1 : 2, and 1 : 3) was prepared by the melt fusion (MF) method. The precise amount of IBP and the amount of the chosen polymer determined based on its apparent solubility were placed in a beaker and heated to about 80°C using a heating mantle. Immediately after melt mixing, the beaker was kept in an ice bath and continuously stirred. 10% colloidal silicon dioxide (AEROSIL® 200) was added as an adsorbent. Then, the mixtures were dried with a vacuum desiccator. The final formulation of ASD-IBP in MF was also produced using the freeze-drying (FD) method to examine the impact of drying on the physicochemical parameters of ASD-IBP. Concisely, the polymer ratio and crystalline IBP in the final formulation prepared by MF were accurately measured, mixed with tert-butyl alcohol, and subsequently frozen at −80°C to produce ASD-IBP using the FD method. The frozen samples were subjected to lyophilization for 24 h using an Eyela FD-1000 freeze dryer (Tokyo Rikakikai, Tokyo, Japan) at a pressure of 15 Pa. The solvent trapped in the freeze dryer was maintained at −50°C.

### 2.5. Equilibrium Solubility Study

The equilibrium solubility of IBP samples was determined using a modified version of a method developed by Higuchi and Connors [21]. An excess amount of IBP and ASD-IBP samples (approximately 50 mg) was placed in a test tube containing 10 mL of distilled water and stored at 37°C for 24 h in an automated water bath shaker. After 24 h, samples of the experimental solutions were obtained, subjected to centrifugation at 10,000 rpm for 5 min, and diluted using methanol. The IBP content was analyzed using the approach outlined in Section 2.2.

### 2.6. Surface Morphology

Scanning electron microscopy (SEM) techniques were employed to investigate the surface morphology of crystalline IBP and ASD-IBP samples (FESEM JEOL JSM-7600F, Singapore). Magnetron sputtering apparatus (MSP-1S, Vacuum Device, Ibaraki, Japan) was used to coat the samples with platinum after using double-sided carbon tape to adhere them to an aluminum sample holder.

### 2.7. X-Ray Powder Diffraction (XRPD)

The X-ray diffraction patterns of IBP and ASD-IBP samples were recorded using a Rigaku X-ray diffractometer (Tokyo, Japan). The instrument emitted Cu K radiation at a current of 30 mA and a voltage of 40 kV. The samples underwent scanning at 2 short-range angles ranging from 5° to 35°, with a step size of 0.2° and a scanning speed of 4° per min.

### 2.8. Differential Scanning Calorimetry (DSC)

To examine the thermal characteristics of IBP and ASD-IBP samples, 3 mg of each sample was introduced into aluminum pans. Subsequently, the samples were subjected to a heating rate of 5°C per minute using a differential scanning calorimeter (DSC) manufactured by Netzsch in Germany. Nitrogen gas (50 mL) was continuously purged throughout the experiment. The calibration standard for the system consisted of indium, which was 99.999% pure and weighed between 8 and 10 mg. The onset temperature for this calibration standard was measured to be 156.6°C.

### 2.9. Thermal Analysis

Thermogravimetric analysis (TGA) analysis was accomplished under a 20 mL/min nitrogen constant flow using a thermogravimetric analyzer system model: (STA 449 F1 Jupiter, NETZSCH, Germany). Accurately weighed amount (5 mg) of crystalline IBP, physical mixture, and ASD-IBP samples were placed in a sealed alumina crucible. All test samples were subjected to a constant heating rate of 10°C/min in the temperature range of 30°C–300°C using an empty alumina pan as reference.

### 2.10. Particle Size Distribution

The mean hydrodynamic diameter of ASD-IBP samples suspended in water was assessed using a Zetasizer ultra instrument (MALVERN, Worcestershire, UK) equipped with a dynamic light scattering (DLS) technique. The measurements were conducted at a temperature of 25°C and an angle of 90°, employing the correlation of photons from light scattering to ascertain the average diameter. The experiment was repeated on all three of them.

### 2.11. Brunauer, Emmett, and Teller (BET) Surface Area Analysis

After degassing the IBP and ASD-IBP samples in a flowing nitrogen atmosphere at 40°C for 14 h, the BET surface area of the samples was evaluated using nitrogen gas adsorption at the ASAP2020 automated adsorption equipment (Micrometric Ltd., USA). The surface area was determined using the Brunauer, Emmett, and Teller (BET) equation for a relative pressure range of 0.01–1.0.

### 2.12. Fourier-Transform Infrared Spectroscopy (FTIR)

FTIR analysis was conducted to ascertain the probability of hydrophobic interactions between the polymers and the substrate. The samples were individually placed on the instrument's sample platform (Perkin Elmer, L160000A, USA), and infrared spectra ranging from 4000 (600 cm^−1^) were acquired using spectrum 10 software. During the analysis, the baseline of each sample was adjusted and normalized. The resulting spectra were smoothed using a nine-point smoothing algorithm.

### 2.13. Formulation of IBP Sustained-Release Matrix Tablets

The precise ASD-IBP (ASD3) was selected based on the earlier study to formulate sustained-release matrix tablets. A direct compression method prepared sustained-release matrix tablets containing ASD-IBP equivalent to 100 mg of IBP. ASD granules were mixed with different amounts of Kollidon® SR (10%, 20%, 30%) and Eudragit® RSPO (10%, 20%, 30%) as a sustained-release polymer. StarTab® was used as a tableting agent, and talc and magnesium stearate were used as a glidant and lubricant, respectively. After mixing, flowability characterization tests were performed to analyze the powder mixture, which was then directly compressed into tablets in a single rotary tablet press (Emtech, USA) utilizing a 12 mm punch. After selecting the suitable ratio of ingredients in matrix tablets, the ASD3-FD was employed in tablet preparation to compare the impact of drying on the performance of tablet characteristics.

### 2.14. Precompression Evaluation of Powder Blends

To evaluate homogenous powder blends, several precompression parameters named loose bulk density, tapped bulk density, Carr's compressibility index, Hausner's ratio, and angle of repose were determined before compression [22].

### 2.15. Postcompression Evaluation of Formulated Tablets

Postcompression parameters such as weight variation, hardness, and friability were determined. Twenty tablets of each formulation were considered for the weight variation test using an electronic balance (Electrolab India Pvt. Ltd.). Also, the hardness and friability of the formulated tablets (six tablets of each formulation) were evaluated using an automatic tablet hardness tester (YD-1 Tablet Hardness Tester, Wincom China) and a friabilator (Electrolab India Pvt. Ltd.), respectively [23].

### 2.16. Dissolution Studies

#### 2.16.1. Dissolution Study of ASD-IBP Samples

Dissolution tests for ASD-IBP samples were carried out for 1 h at 37°C using 50 mL of pH 6.8 phosphate buffer with constant stirring of 75 rpm by a magnetic stirrer. The experiment involved obtaining samples of 1 mL at certain time intervals (5 min, 10 min, 15 min, 20 min, 30 min, 45 min, and 60 min). These samples were subsequently subjected to centrifugation at 10,000 rpm for 5 min. Following centrifugation, the samples were diluted using a phosphate buffer with a pH of 6.8. The spectrophotometric measurement of IBP concentration was conducted at a wavelength of 221 nm.

#### 2.16.2. Dissolution Study of Sustained-Release IBP Matrix Tablets

Dissolution experiments for sustained-release IBP matrix tablets were conducted at 37°C for 12 h using the USP paddle method at 50 rpm in 900 mL of pH 6.8 phosphate buffer with the dissolution tester system electrolab dissolution tester, Electrolab India Pvt. Ltd. At predetermined intervals, 5 mL samples were withdrawn from the dissolution vessel followed by quick addition of 5 ml fresh medium to maintain a constant volume. After filtering through a 0.45 *μ*m membrane, the samples were diluted with phosphate buffer at pH 6.8. Spectrophotometry was used to determine the IBP concentration. All experiments were run in triplicate.

### 2.17. Dissolution Kinetics

#### 2.17.1. Model-Independent Fit Factors

The dissolution profiles of various formulations of IBP and a reference sample (coded as REF) were compared using model-independent methods and dissolution efficiency (DE). Model-independent methods compare the two profiles solely at observed time points. This method employs both the difference factor (*f*_1_) and the similarity factor (*f*_2_). The difference factor (*f*_1_) quantifies the percentage difference between the two curves (reference and test samples) at each time point and calculates the relative error between the two curves. The similarity factor (*f*_2_) is a logarithmic reciprocal square root modification of the sum of squared error that measures the degree of similarity (%) between two curves. The difference factor (*f*_1_) and similarity factor (*f*_2_) were determined using the established equations (24). Mean dissolution time (MDT), as an example of a model-independent factor, is determined from the cumulative curves of dissolved IBP as a function of time [25]. Moreover, the dissolution efficiency (DE), which is the area under the dissolution curve within a period, was also calculated [24]:

#### 2.17.2. Model-Dependent Dissolution Kinetics

Numerous model-dependent mathematical models, such as zero-order, first-order, Higuchi, Hixson–Crowell, Korsmeyer–Peppas, and Weibull, were utilized to investigate the *in vitro* release kinetics. The following equations describe the model-dependent mathematical kinetics [24]. The best-fitting equation uses the coefficient of determination (*R*^2^), adjusted coefficient of determination (*R*_adjusted_^2^), and Akaike information criterion (AIC) [26]. According to the previously defined criteria for selecting the best kinetic model, the best and most accurate models should be selected using appropriate metrics such as *R*^2^, *R*_adjusted_^2^, and AIC. The optimal model may be evaluated with lower AIC and higher *R*_adjusted_^2^ values.

### 2.18. Statistical Analysis

The data are presented as the mean and standard deviation (S.D.). The diagrams were generated utilizing GraphPad, Prism 8.0 software (GraphPad Software, LaJolla, CA). The mathematical parameters were computed using the DDSolver programme [27]. A one-way analysis of variance (ANOVA) was used to compare means with pairwise comparisons using Fisher's least significant difference technique, which was employed for statistical comparisons. In all analyses, a *p* value less than 0.05 was considered significant.

## 3. Results and Discussion

Scientists continuously struggle with poorly soluble pharmaceuticals in the emerging formulation development and drug delivery field. Fortunately, SD has emerged as a promising strategy for combating the challenges of drugs with poor solubility [28]. SDs usually have two parts: the carrier and the API. Choosing a suitable carrier is essential for the best drug release and therapeutic results [29]. Several investigations have shown that the synergistic effect of carriers in SDs may result in head-to-head contacts and electrostatic interactions [30]. This occurs due to hydrophilic groups bound to the surface by cohesive forces, which reduce surface tension, forming an inner hydrophobic core and increasing solubility [31–33]. Therefore, the current study aimed to use an amphiphilic carrier to prepare and characterize ASD-IBP to improve biopharmaceutical properties.

### 3.1. Selection of Polymers for ASD System

Choosing polymers as carriers is critical in improving the biopharmaceutical characteristics of poorly soluble drugs within the self-dispersing system. Excipients experiencing increasing popularity are amphiphilic polymers, characterized by hydrophilic and lipophilic groups. Encapsulation facilitates the formation of polymeric micelles by promoting interactions between lipophilic groups of amphiphilic polymers and weakly soluble pharmaceuticals. These micelles possess a hydrophobic core and a hydrophilic outside, enhancing the solubility of drugs with low solubility. This phenomenon has been observed in previous studies [34].

**Figure 1:**
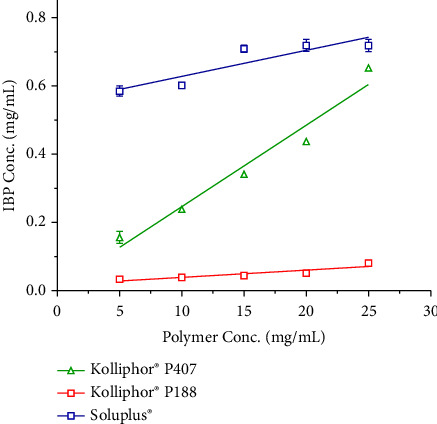
Apparent solubility of IBP in the aqueous solution of Soluplus®, Kolliphor® P188, and Kolliphor® P407 at various concentrations (5–25 mg/mL). Data presented as mean ± S.D. (*n* = 3).

The investigation focused on determining the observed solubility of IBP in water when various concentrations of predissolved polymers, such as Soluplus®, Kolliphor® P188, and Kolliphor® P407, ranging from 5 to 25 mg/mL, were present. The aim was to select appropriate polymers for the IBP amorphous solid dispersion (ASD) system. Soluplus®, Kolliphor® P188, and Kolliphor® P407 are frequently employed as carriers with amphiphilic characteristics to improve the solubility of poorly soluble drugs due to their biocompatibility and widespread availability in the commercial market [35–37]. As shown in Figure 1, the aqueous solubility of IBP increased as the polymer concentration increased. All analyzed polymers exhibited a linear (*A*_*L*_-type) relationship between increasing IBP solubility and polymer concentration. Soluplus® and Kolliphor® P407 enhanced the solubility of IBP significantly (*p* < 0.01) compared to Kolliphor® P188. The aqueous solubility of IBP was increased by 13.5-fold and 12.3-fold with the addition of Soluplus® and Kolliphor® P407 at a 25 mg/mL concentration. Similarly, Kolliphor® P188 improved the solubility by 1.5-fold at the same concentration. The increased dispersibility and miscibility of IBP disseminated in polymers led to an increase in the apparent solubility of IBP in amphiphilic block copolymers [38, 39]. Furthermore, the stability constants (Ks) were determined by a linear regression analysis of the generated phase solubility diagram. The obtained data have been compiled and are displayed in Table 1.

The *K*_*s*_ is significantly (*p*=0.0031) higher for Kolliphor® P407 than Kolliphor® P188, justifying the positive effect on solubility enhancement of IBP. Although higher *K*_*s*_ for Soluplus®, due to the lower melting point of Kolliphor® P407, it was chosen as the suitable polymer for the melt fusion method. These findings implied that IBP might interact with Kolliphor® P407 more successfully and entangled in the micelle's hydrophobic core, improving its solubility. Thus, Kolliphor® P407 was chosen as the carrier for developing ASD-IBP to improve the physicochemical behavior of IBP based on its apparent solubility and kinetic data.

### 3.2. Selection of an Appropriate Ratio of Polymer

To identify the ideal ratio for further study, the physicochemical characteristics, including equilibrium solubility and particle size of ASD-IBP, which was prepared by melt fusion using different ratios of IBP and Kolliphor® P407, were investigated to determine the optimal ratio for further study. Considering the limited aqueous solubility of crystalline IBP (50.29 *µ*g/mL), all formulations (ASD1-ASD4) enhanced IBP solubility. The equilibrium solubility of the ASD-IBP formulations is shown in Table 2. ASD3 and ASD4 demonstrated the greatest solubility enhancement among all formulations.

IBP solubility increased significantly (*p* < 0.0001) with the double and triple amount of Kolliphor® P407, reaching 1,421.15 ± 24.73 *µ*g/mL and 1,969.69 ± 32.08, respectively. Kolliphor® 407 is a block copolymer composed of polyoxyethylene–polyoxypropylene–polyoxyethylene (PEO-PPO-PEO) chains, which solubilize and emulsify substances. Kolliphor® P407 is composed of hydrophobic poly (propylene oxide) (PPO) blocks and hydrophilic poly (ethylene oxide) (PEO) blocks, which serve as surfactants (38). The critical micelle concentration (CMC) of Kolliphor® P407 is 34.2 mg/L [40]. This suggests that combining these two biocompatible polymers may contribute to the enhanced dissolution behavior and oral absorption of IBP. Moreover, it was reported that polymeric micelles dissociate very slowly due to their kinetically stable nature [41].

To investigate the micellization capability of Kolliphor® P407, the mean particle size of the ASD formulations was also evaluated, and the results are presented in Table 2. According to the micellar size distribution, the size of micelles plays a considerable role in how they enter different cells *in vivo*, regardless of the route of administration [42]. According to the results of micellar size distribution, ASD1 and ASD3 showed smaller micelle sizes with better colloidal stability at room temperature. From ASD3 and ASD4, there was no statistically significant difference, and it was evident that low polymer concentration is always preferable in the industry for better manufacturability and scalability. Considering the enhanced solubility and particle size distribution, formulation ASD3 was selected for further physicochemical characterization. Moreover, with the same polymer-drug ratio as ASD3, optimized ASD formulation prepared by freeze-drying (ASD3-FD) was also characterized to assess the drying method's influence on the physicochemical characteristics of the formulations.

### 3.3. Physicochemical Characterizations

**Figure 2:**
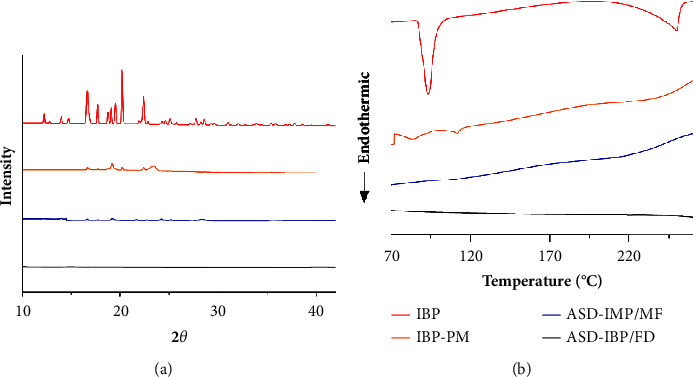
Crystallinity assessment of IBP samples using (a) XRPD and (b) DSC.

Amorphous forms can have more excellent solubility and dissolution rates than crystalline forms due to their higher energy state. Consequently, determining crystallinity becomes crucial for establishing product quality. XRPD and DSC analyses were utilized to investigate the crystalline structure of ASD-IBP (Figure 2). The XRPD pattern of crystalline IBP revealed several pointed peaks, with the most prominent peak located at approximately 20.18° (Figure 2(a)), indicating the crystalline nature of IBP. In contrast, ASD-IBP/MF and ASD-IBP/FD exhibited a diffractive halo pattern. Minimal peaks were detected in the diffractogram for ASD-IBP, indicating that IBP was in an amorphous state. In DSC analysis, although crystalline IBP had an endothermic peak around 100°C (Figure 2(b)), the endothermic peak at the melting point of crystalline IBP was absent in ASD-IBP/MF and ASD-IBP/FD.

**Figure 3:**
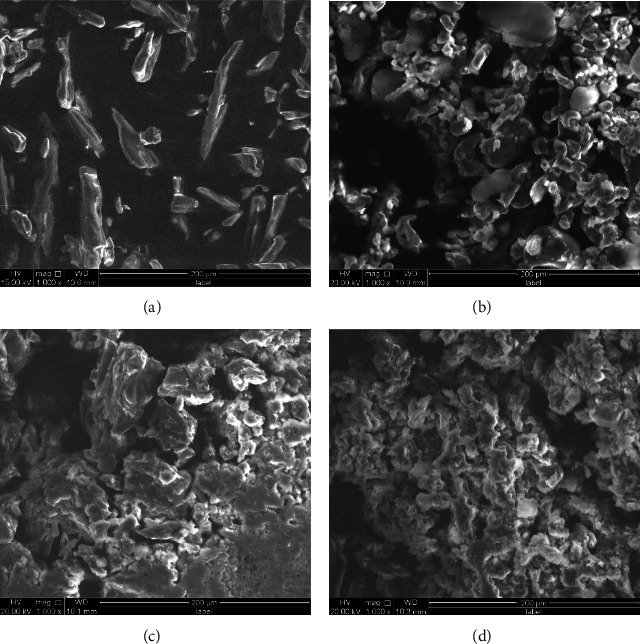
Microscopic images observed by scanning electron microscope: (a) crystalline IBP, (b) physical mixture, (c) ASD-IBP/MF, and (d) ASD-IBP/FD.

The elevated level of free energy observed in the amorphous state can potentially confine the drug molecule within an amorphous solid dispersion (ASD), hence impeding drug precipitation or recrystallization in the supersaturated state. This characteristic offers a notable advantage in augmenting lipophilic medicines' solubility [43]. According to XRPD and DSC analyses, the amorphization of IBP during the preparation procedure resulted in superior dissolving properties. Using SEM observations, the surface morphology of the IBP samples was evaluated (Figure 3). The morphology of crystalline IBP consisted primarily of irregularly shaped, disseminated particles (Figure 3(a)). ASD-IBP/MF and ASD-IBP/FD, on the other hand, appeared conventional and flaky (Figures 3(c) and 3(d)). Compared to the crystalline IBP, ASD-IBP particles were homogenous, and their size was considerably reduced. The findings revealed that integrating IBP into the polymer matrix was successful, suggesting a high degree of assimilation. In contrast to crystalline IBP, the SEM micrographs reveal a notable augmentation in the surface area of ASD-IBP in the freeze-drying process (Figure 3(d)). According to the Noyes–Whitney equation, the increased particle surface area produced by micronization is a significant factor in accelerating dissolution [44].

In addition, the BET study revealed an increase in the BET surface area and total pore volume in ASD-IBP/MF and ASD-IBP/FD compared to crystalline IBP. Besides, the BET study also revealed a reduction in the BET surface area and total pore volume in ASD-IBP/FD compared to ASD-IBP/MF. ASD-IBP/MF and ASD-IBP/FD manifested a type IV adsorption isotherm. Alternatively, the average pore diameter was slightly reduced from 53.36 (ASD-IBP/MF) to 49.88 (ASD-IBP/FD), as shown in Table 3.

On the other hand, the size distribution of polymeric micelles is believed to be one of the most critical factors in enhancing the biopharmaceutical properties of a drug [38]. This study evaluated the micellization capabilities of ASD-IBP using the DLS analysis. The DLS analysis was conducted on water-dispersed samples of ASD-IBP, which resulted in the observation of nanoparticle formation. The average particle size was 261 nm for ASD-IBP/FD, with a polydispersity index (PDI) of 0.258. Similarly, ASD-IBP/MF exhibited an average particle size of 305.2 nm, with a PDI of 0.215. Furthermore, the hydrophilic chain on the surface of polymeric micelles contributes to enhanced drug solubility, dispersibility, and diffusivity within the mucus layer. Consequently, this phenomenon promotes efficient drug absorption after oral administration [45].

As a result, SEM and DLS data images confirmed the absence of crystalline IBP during the ASD-IBP preparation process. These advantages may contribute to the improved dissolution behavior of IBP.

### 3.4. Thermal Behavior of IBP Samples

**Figure 4:**
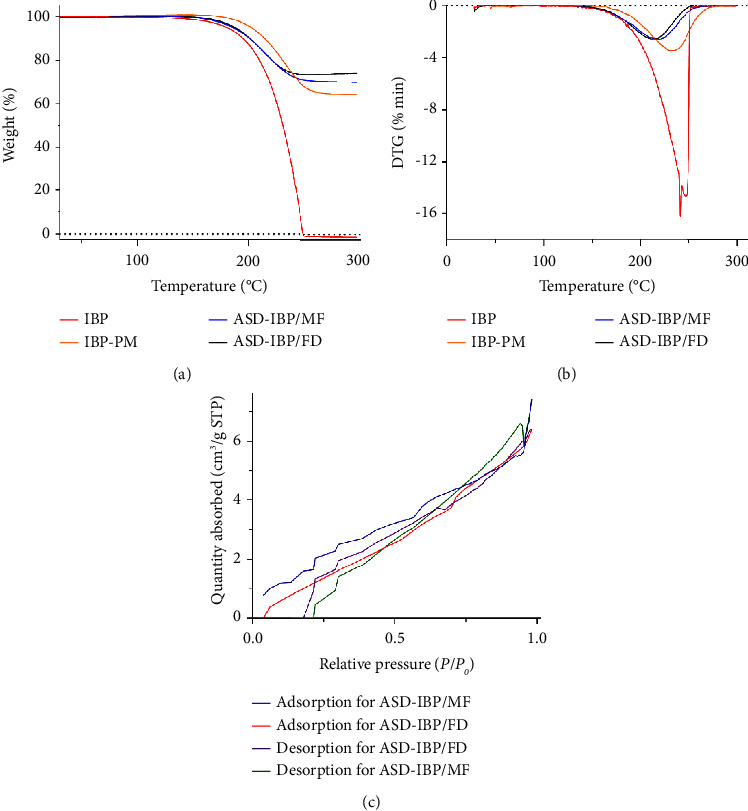
Thermal behavior of various IBP samples: (a) TGA curves, (b) DTG curves, and (c) N_2_ adsorption-desorption isotherms of IBP samples.

Thermal analyses were used to investigate the effects of thermal stress on the active substances. These analyses provided three different pieces of information about a particular phase transition: (1) the identity of the involved phase (structural information), (2) the temperature of the phase transition, and (3) the heat capacity of the system [46]. The temperature rise could cause thermal stress, which could occasionally cause the API's structural changes. To investigate the thermal behavior of IBP samples, TGA/DTG tests were carried out.  Figure 4 shows the TGA and DTG curves of the IBP samples. The TGA curves demonstrated that the crystalline IBP thermally degraded between 200°C and 300°C. The samples showed thermal stability up to 150°C, indicating less moisture content in the sample, then melting and peaking at 240°C. The TG/DTG curves show that the thermal decomposition takes place in one step for all samples.

Moreover, the DTG analysis showed that the crystalline IBP had deteriorated between 200°C and 300°C. Ibuprofen was pure on the thermal analysis diagram, with one endothermic peak around 100°C corresponding to its melting point [47], and decomposing in one step between 200°C and 300°C. It was observed that 99.9% of the mass was lost at temperatures between 250°C and 300°C. The ASD-IBP simultaneously indicates degradation across a wide temperature range. With ASD-IBP, the overall mass loss percentage was estimated to be around 70%. The mass loss percentage of ASD-IBP/MF and ASD-IBP/FD in the same temperature range is similar, according to DTA/TGA diagrams of the samples. However, the mass loss percentage of IBP/PM is more significant than ASD-IBP. This distinction can result from the complex formation between IBP and polymer. The likelihood of a chemical interaction between IBP and the polymer while creating ASD is another significant result of heat analysis. The information suggests that IBP and Kolliphor® P407 may interact chemically to produce a complex in ASD.

### 3.5. Drug Polymer Interactions

**Figure 5:**
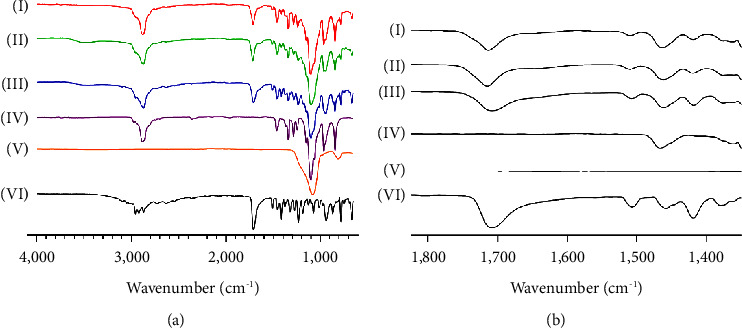
Drug-polymer interaction studies of IBP samples using FT-IR. Baseline-corrected and normalized IR data of IBP samples in the spectral wavenumber region from (a) 4,000–600 cm^−1^ and (b) 1,800−1,300 cm^−1^. (i) ASD-IBP/FD, (ii) ASD-IBP/MF, (iii) PM, (iv) Kolliphor® P407 (v) AEROSIL, and (vi) crystalline IBP.

In theory, amorphous molecules can be dispersed at a molecular level within the matrix carrier of a solid dispersion (SD) formulation. The intermolecular interaction among these molecules has the potential to lead to an improved amorphization of the medication [48]. The FTIR analysis determined the molecular state of crystalline IBP and processed ASD-IBP.  Figure 5 illustrates the FTIR spectrum. The presence of the asymmetric C-H stretching vibration was identified by a distinct and well-defined infrared absorption band at 2,954.25 cm^−1^ and 1,705.31 cm^−1^ (carbonyl-stretching of the isopropionic acid group) (Figure 5). It is believed that the IR spectrum patterns of IBP samples reflect diverse chemical environments. The diagram (Figure 5) depicts the absorption maxima at 2,878.4 cm^−1^ (C-H stretch aliphatic), 1,341.93 cm^−1^ (in-plane O-H bend), and 1,144.5 cm^−1^ (C=O stretching) in the IR spectra of Kolliphor® P407.

In contrast, the lack of a distinct peak in the IR spectra of ASD-IBP/MF and ASD-IBP/FD indicates negligible electrostatic interaction. The FTIR spectrum analysis demonstrated that the inclusion of Kolliphor® P407 is associated with a negligible hydrophobic interaction that is unlikely to impact the chemical composition of IBP, as indicated by the findings of the FTIR spectrum analysis. Theoretically, this phenomenon is desirable because of the potential for drug-polymer interactions to prolong the dissolving process effectively. The dissolution process will exhibit a greater thermodynamic driving force when there are weak or no interactions between the drug and polymer [49, 50].

### 3.6. Dissolution Behavior of ASD-IBP Samples

**Figure 6:**
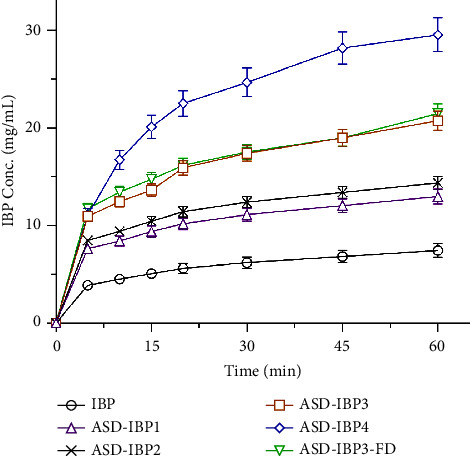
Dissolution tests of IBP samples in pH 6.8 phosphate buffer. Data represent the mean ± S.D. of 3 experiments.

Dissolution of IBP in pH 6.8 phosphate buffer was improved with the increment in the ratio of Kolliphor® P407 in ASDs (Figure 6).

Enhanced dissolution of IBP in ASDs from crystalline IBP may be attributable to the surface activity, hydration effect, and solubilizing effect of Kolliphor® P407. This may result in reduced agglomeration and increased surface area [2]. However, the drug release pattern shown in Figure 6 indicates insignificant differences between the ASD-IBP prepared by MF and FD.

### 3.7. Precompression Evaluation of Powder Blends

The precompression parameters for all seven formulations (the composition of the formulations is presented in Table 4) were performed, and the results are presented in Table 5. The powder is free-flowing if the Hausner ratio is less than 1.25, whereas a ratio of more than 1.25 indicates poor flowability. The better the flow properties, the lower the Carr's Index. For example, a range of 5–10 means excellent flow, 11–15 good flow, 16–20 fair flow, and >23 poor flow. However, an angle of repose within 35 degrees suggests good flow properties [22].

The bulk density and tapped density of the prepared blend ranged from 0.226 ± 0.027 to 0.561 ± 0.033 g/mL and 0.279 ± 0.034 to 0.637 ± 0.017 g/mL, respectively. The Carr's index was found to range from 7.92 ± 1.331 to 18.87 ± 1.247%, and the Hausner's ratio ranged from 1.09 ± 0.035 to 1.23 ± 0.019. The angle of repose was found to be ranging from 24.23 ± 0.035° to 34.99 ± 0.042° for all formulations. From the results of precompression evaluation tests, the powder blend of all the formulations has good flowing properties and good compressibility, and the results are within the pharmacopeial limit [22]. However, based on precompression parameters shown in Table 5, it can be concluded that ASD-IBP prepared by melt fusion (F3) showed better-flowing properties compared to ASD-IBP prepared by freeze-drying (F3-FD).

### 3.8. Postcompression Evaluation of Formulated Tablets

The result of the postcompression parameters of all seven formulations is shown in Table 6. Weight variation is used to indicate the uniformity of the tablet content. It is found that from all formulations included in the study, formulation F6 had the most negligible average weight of 592.00 ± 12.31 mg, and formulation F3-FD had the highest average weight of 605.00 ± 19.03 mg. But, all formulations of ASD-IBP tablets showed acceptable uniformity of weight as the weight of the tablets was 600 mg; hence, the excellent weight variation range was between 570 and 630 mg (±5%) as stipulated by the USP [23].

The mechanical properties of pharmaceutical tablets are measurable by their friability, hardness, or crushing strength [23]. The hardness test determines the ability of tablets to endure stress or pressure during handling, packaging, and transportation [24]. The hardness of all formulations was found to be in the range of 3.50 ± 0.37 (F4)–6.19 ± 0.58 (F3) kg/cm^2^. This study found that hardness was higher for the tablets (F1, F2, F3) containing Kollidon® SR as release retardant compared to the tablets (F4, F5, and F6) containing Eudragit® RSPO. Besides, hardness was increased with the increment of Kollidon® SR ratio, that is why formulation F3 (30% Kollidon® SR) showed the highest hardness in this study. Moreover, tablets formulated using ASD-IBP prepared by MF (F3) showed higher hardness compared to tablets formulated using ASD-IBP prepared by freeze drying (F3-FD).

The hardness test may not be the finest indicator of tablet behavior during packaging and handling. Loss due to abrasion or tablet friability measurement may be a more pertinent parameter [24]. This investigation revealed that all formulations had friability values ranging from 0.21 ± 0.019% to 0.45 ± 0.073%, indicating that all formulations met the pharmacopeial specification for friability, which specifies that weight loss of not more than 1% is deemed generally acceptable [23]. The results of friability identified that the formulated tablets were mechanically stable and had good integrity of the tablet.

### 3.9. Dissolution Behavior of Sustained-Release Matrix Tablets

**Figure 7:**
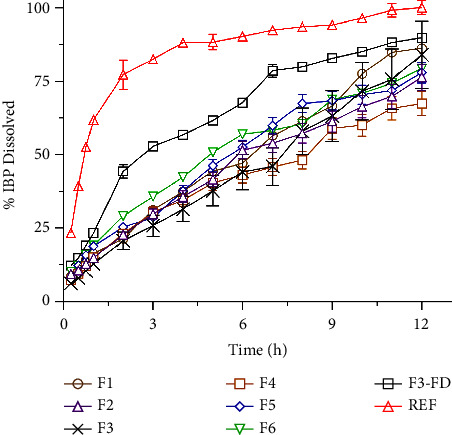
*In vitro* cumulative (%) drug release profile of various formulations of ASD-IBP/SR tablets. Data represent the mean ± S.D. of 3 experiments.

To understand the drug release profile from solid dispersed tablets, IBP *in vitro* dissolution study was carried out for up to 12 h in phosphate buffer (pH 6.8). Then, the cumulative percentage of drug release was determined from the standard calibration curve and fitted into many mathematical models to obtain an idea of the drug release profile from the formulations. The cumulative percentage of drug released vs. time plot is shown in Figure 7 for all formulations and reference samples. The cumulative percent drug release for all formulations after 12 h of dissolution was found to be within the range of 69.43 ± 4.11% (F4)–87.89 ± 1.11% (F3-FD), and for the reference sample, it was 100.10 ± 2.34% (REF). As evidenced by several studies, tablet hardness can affect the porosity and permeability of the tablet matrix, which in turn influences the diffusion of the drug out of the tablet [51]. Therefore, harder tablets may have a denser structure, leading to slower drug release rates. Among the formulations, F3 has the better hardness (6.19 kg/cm^2^), which may lead to a slower release of IBP as MDT was found 5.6 h. Thus, based on the dissolution and hardness tests, F3 formulation was chosen as the optimized formulation. With the same ratio of excipients, tablets formulated using ASD-IBP prepared by FD (F3-FD) exhibited identical dissolution behavior. Though the formulations containing ASD-IBP prepared by MF and FD methods have identical dissolution behavior, the pre and postcompression characters differ between the drying approaches. The difference in pre and postcompression parameters between MF and FD might be due to the agglomeration of particles or collapse of the porous structure in the FD process, affecting the flowability and compressibility of the powder [52]. Hence, ASD prepared by MF, can produce particles with excellent uniformity and controlled particle size distribution, improving compressibility and tablet uniformity. In addition, Kollidon® SR showed better release retardant properties than Eudragit® RSPO. Kollidon® SR is spray-dried polyvinyl acetate containing soluble polyvinylpyrrolidone in an 8 : 2 ratio. Kollidon® SR is a suitable polymer for fabricating sustained-release matrix tablets generated through direct compression due to its outstanding flowability and compressibility [53].

On the other hand, Eudragit® polymers are well-known for their sustained-release and solubilizing capabilities, as they are derivatives of polymethacrylic acid-co-methyl methacrylate. Eudragit® RSPO is insoluble at physiological pH due to the existence of low-level quaternary ammonium groups. Still, it can swell and hence could be a suitable carrier for sustained release delivery [54].

### 3.10. Dissolution Kinetics

#### 3.10.1. Model-Independent Fit Factors

A model-independent approach of difference factor (*f*_1_) and similarity factor (*f*_2_) was used to demonstrate the equivalence of all the formulated tablets and the reference product. In this study, the difference factor (*f*_1_) and similarity factor (*f*_2_) were calculated for all seven formulations of ASD-IBP tablets by using a reference sample (Bufen® SR, 300 mg capsule, Drug International Ltd., Bangladesh). The *f*_2_ value was found to vary from 17.1 for F3 to 28.35 for F3-FD. The *f*_1_ value ranged from 31.982 for F3-FD to 56.36 for F3 (Table 7). When comparing two dissolution profiles, *f*_1_ should be between 0 and 15, while f2 should be between 50 and 100 to ensure sameness of the two dissolution profiles [24]. Thus, the dissolution profiles of formulated tablets were significantly (*p* < 0.01) different from those of the reference product. The notable disparity in the dissolution profiles of the formed tablets and the reference product raises concerns regarding the sameness of the two formulations, which might affect the product's bioavailability, bioequivalence, safety, efficacy, quality, and consistency.

In addition, the release profiles were also compared by calculating DE for various formulated tablets included in the study. The DE value was found to vary from 38.57% for F3 to 60.93% for F3-FD, whereas 86.20 for the REF (Table 7). Therefore, it is revealed that tablets containing ASD granules prepared by melt fusion and freeze-drying differ in the DE value. However, if the difference in their dissolution efficiency falls within acceptable ranges (±10%), the reference and test products can be considered equivalent [55]. However, all formulated tablets were far from the established limit (±10%).

The mean dissolution time (MDT) reflects the release of the drug from the dosage form and quantifies the effectiveness of the polymer in slowing down this process. A greater MDT suggests a slower rate of drug release from the dosage form [55]. This leads to the slow onset of action and higher drug-retaining ability of the polymer and vice versa. The MDT value ranged from 3.6 h for F3-FD to 5.6 h for F3, whereas it was 1.9 h for the reference product (Table 7). Tablets prepared with Kollidon® SR showed higher MDT values than those designed with Eudragit® RSPO. Moreover, tablets formulated using ASD-IBP prepared by MF (F3) showed higher MDT values than those developed using ASD-IBP prepared by FD (F3-FD).

#### 3.10.2. Model-Dependent Dissolution Kinetics

The dissolution profiles of all formulated tablets and the reference product were determined by fitting experimental data to model-dependent mathematical models, such as zero-order, first-order, Higuchi, Hixson–Crowell, Korsmeyer–Peppas, and Weibull. As indicated in Table 8, the regression parameters of all the samples, such as correlation coefficients and rate constants, were calculated and compared. The model with the highest adjusted determination coefficient (*R*_adjusted_^2^) and the lowest Akaike information criterion (AIC) best fit the release data [25, 56].

As shown in Table 8, based on the highest value of *R*_adjusted_^2^ and the smallest AIC values for all dissolution data of all the formulated tablets and reference products, among the six models fitted to each dissolution profile, the Weibull model was best fitted to the dissolution data of the formulations (F2, F5, F3-FD, and REF) and the formulations (F4 and F6) and was best fitted to the Korsmeyer–Peppas model. On the other hand, for formulations F1 and F3, the best-fitted model was the zero-order kinetics based on the highest value of *R*_adjusted_^2^ and the Weibull model based on the lowest value of AIC value. However, there is no statistically significant difference in the *R*_adjusted_^2^ value of the formulations (F1 and F3) in terms of zero-order kinetics and Weibull model. For this reason, it can be declared that the Weibull model is predominant among all the formulated tablets and reference product under investigation.

The dissolution data were fitted into the Korsmeyer–Peppas model to confirm the diffusional mechanism. As indicated in Table 8, the diffusion coefficient (*n*) found for all the formulated tablets were more than 0.5, whereas for the reference product, it was less than 0.5. A diffusion coefficient value (*n*) of less than 0.5 is consistent with a diffusion-controlled release or Fickian diffusion. In contrast, values of *n* between 0.5 and 1 indicate non-Fickian (anomalous) release mechanisms [57]. Moreover, in this study, the diffusion coefficient (*n*) was within the range of 0.52–0.74 for all the formulated tablets, indicating a non-Fickian diffusion mechanism. Diffusion and matrix erosion were, therefore, responsible for controlling drug release. Thus, the drug release was regulated by multiple procedures for all formulated matrix tablets. On the other hand, the diffusion coefficient (*n*) was 0.24377 for the reference product, indicating a Fickian diffusion.

## 4. Conclusion

The therapeutic applicability of IBP is limited due to its poor solubility with pH dependency, low oral bioavailability, and short elimination half-life. In this study, IBP was prepared as an ASD utilizing two commercially viable techniques: solvent evaporation by rotary vacuum drying (RVD) and freeze-drying. The solubility and dissolution characteristics of IBP were significantly improved in all of the formulated solutions. In addition, a sustained-release matrix tablet formulation with the precise amount of ASD-IBP was prepared. The results indicate that the formulated product demonstrated dissolution profiles appropriate for prolonged-release properties. Therefore, the combination of the ASD approach and the sustained-release idea offers considerable promise in improving the biopharmaceutical performance of IBP.

## Figures and Tables

**Table 1:** Apparent solubility data of complexes of IBP with different polymers.

Complex/parameter	*S*_0_ (mg/mL)	Slope	*R* ^2^	*K*_*s*_	C.E.
IBP-Kolliphor® P407	0.05329	0.007651	0.7846	29.39105	0.00777
IBP-Kolliphor® P188		0.002151	0.8207	8.308788	0.00216
IBP-Soluplus®		0.02381	0.9564	89.9759	0.025001

*S*_0_: solubility of IBP in water; *K*_*s*_: stability constant; C.E.: complexation efficiency.

**Table 2:** The effect of different drugs: polymer ratios on the solubility and particle size distribution.

	Ratio (IBP : KP 407)	Equilibrium solubility (*µ*g/mL)	Particle size distribution		
			Mean diameter (nm)	PDI	ZP (±mV)
Crystalline IBP		50.29 ± 2.20			
ASD1	2 : 1	325.05 ± 17.66	144.8	0.422	−50.5
ASD2	1 : 1	616.71 ± 16.39	741.6	1.019	−42.9
ASD3	1 : 2	1,421.15 ± 24.73	305.2	0.215	−57.4
ASD4	1 : 3	1,969.69 ± 32.08	950.3	0.802	−43.9

IBP: ibuprofen; KP 407: Kolliphor® P407; PDI: polydispersity index; ZP: zeta potential.

**Table 3:** Brunauer–Emmett–Teller (BET) surface area of IBP samples.

Parameter	IBP	ASD-IBP/MF	ASD-IBP/FD
BET surface area (m^2^/g)	5.42	8.58	7.90
Average pore diameter, 4 V/S (Å)	36.61	53.36	49.88
Total pore volume (cc/g)	0.0050	0.0114	0.0099

**Table 4:** Formulation of IBP sustained-release matrix tablets.

Ingredients	Formulation code (quantity in mg)						
	F1	F2	F3	F4	F5	F6	F3-FD
ASD3∗ (100 mg eqv. of IBP)	333.33	333.33	333.33	333.33	333.33	333.33	333.33
StarTab®	194.67	134.67	74.67	194.67	134.67	74.67	74.67
Kollidon® SR	60	120	180	—	—	—	180
Eudragit® RSPO	—	—	—	60	120	180	0
Magnesium stearate	6	6	6	6	6	6	6
Talc	6	6	6	6	6	6	6
Total (mg)	600	600	600	600	600	600	600

^∗^For formulation F3-FD, ASD3-FD was used.

**Table 5:** Precompression parameters of powder blends.

Formulation codes	Bulk density (gm/mL)	Tapped density (gm/mL)	Carr's index (%)	Hausner's ratio	Angle of repose (°)
F1	0.495 ± 0.011	0.538 ± 0.016	7.92 ± 1.331	1.09 ± 0.035	25.63 ± 0.012
F2	0.533 ± 0.026	0.579 ± 0.037	8.00 ± 1.205	1.09 ± 0.051	24.23 ± 0.035
F3	0.481 ± 0.021	0.543 ± 0.028	11.54 ± 1.087	1.13 ± 0.026	28.81 ± 0.017
F4	0.538 ± 0.015	0.617 ± 0.022	12.90 ± 1.203	1.15 ± 0.042	30.96 ± 0.030
F5	0.538 ± 0.029	0.610 ± 0.039	11.83 ± 1.046	1.13 ± 0.037	27.69 ± 0.023
F6	0.561 ± 0.033	0.637 ± 0.017	11.96 ± 1.378	1.14 ± 0.032	27.02 ± 0.018
F3-FD	0.226 ± 0.027	0.279 ± 0.034	18.87 ± 1.247	1.23 ± 0.019	34.99 ± 0.042

All values are mean ± S.D.; *n* = 3.

**Table 6:** Postcompression parameters of formulated tablets.

Formulation codes	Weight variation (*n* = 20)	Hardness (*n* = 6)	Friability (*n* = 6)
		(kg/cm^2^)	(% weight loss)
F1	602.99 ± 9.09	3.56 ± 0.26	0.21 ± 0.043
F2	600.49 ± 9.06	5.02 ± 0.30	0.41 ± 0.052
F3	601.99 ± 10.08	6.19 ± 0.58	0.38 ± 0.021
F4	596.50 ± 8.99	3.50 ± 0.37	0.29 ± 0.044
F5	593.83 ± 8.96	3.79 ± 0.41	0.21 ± 0.019
F6	591.34 ± 9.82	4.09 ± 0.16	0.25 ± 0.033
F3-FD	604.32 ± 9.12	4.26 ± 0.41	0.45 ± 0.073

All values are mean ± S.D.

**Table 7:** Various dissolution-related model-independent fit factors of ASD-IBP tablets.

	REF	F1	F2	F3	F4	F5	F6	F3-FD
*f*_1_		45.00	52.99	56.36	53.61	43.23	43.36	31.982
*f*_2_		21.2	18.5	17.1	18.4	22.3	22.6	28.35
MDT (h)	1.9	5.2	4.9	5.6	4.4	4.5	4.3	3.6
DE (%)	86.20	48.59	41.69	38.57	40.93	50.26	50.16	60.93

*f*_1_: difference factor; *f*_2_: similarity factor; MDT: mean dissolution time; DE: dissolution efficiency.

**Table 8:** Determination of dissolution kinetics of different model-dependent release kinetic models.

Model	Parameters	Samples							
		F1	F2	F3	F4	F5	F6	F3-FD	REF
Zero-order	*R* ^2^	0.9953	0.9846	0.9962	0.9768	0.9698	0.9645	0.9179	0.633
	*R*_adjusted_^2^	0.9949	0.9834	0.9959	0.975	0.9674	0.9618	0.9116	0.6048
	*K*_0_	7.67	6.45	6.18	6.18	7.68	7.53	8.94	11.399
	AIC	94.00	96.47	80.92	103.39	107.35	110.84	121.29	149.524

First-order	*R* ^2^	0.9674	0.9838	0.9743	0.9401	0.9767	0.9663	0.9778	0.9118
	*R*_adjusted_^2^	0.9674	0.9838	0.9743	0.9401	0.9767	0.9663	0.9778	0.9118
	*K*_1_	0.13	0.10	0.09	0.09	0.13	0.13	0.19	0.9415
	AIC	88.83	72.15	79.82	88.32	82.04	85.65	84.28	100.72

Higuchi	*R* ^2^	0.9375	0.9656	0.9169	0.9845	0.9709	0.9904	0.9826	0.3694
	*R*_adjusted_^2^	0.9375	0.9656	0.9169	0.9845	0.9709	0.9904	0.9826	0.3694
	*K*_*h*_	21.91	18.57	17.55	17.96	22.23	21.94	26.22	35.125
	AIC	98.59	83.49	97.41	68.06	85.36	66.88	80.64	130.23

Hixon–Crowell	*R* ^2^	0.9752	0.9731	0.9798	0.9168	0.9644	0.9394	0.9449	0.5420
	*R*_adjusted_^2^	0.9752	0.9731	0.9798	0.9168	0.9644	0.9394	0.9449	0.5420
	*K*_*d*_	0.04	0.03	0.03	0.03	0.04	0.04	0.05	0.1304
	AIC	84.73	79.77	76.22	93.27	88.36	94.45	97.93	125.43

Korsmeyer–Peppas	*R* ^2^	0.9904	0.9960	0.9897	0.9923	0.9884	0.9960	0.9832	0.8475
	*R*_adjusted_^2^	0.9897	0.9957	0.9889	0.9917	0.9875	0.9957	0.9819	0.8357
	*K*_kp_	13.54	13.47	9.46	15.59	17.72	19.52	25.29	57.716
	*n*	0.74	0.66	0.80	0.57	0.61	0.56	0.52	0.2433
	AIC	72.50	53.04	68.06	59.58	73.59	55.51	82.08	110.93

Weibull	*R* ^2^	0.9924	0.9970	0.9939	0.9907	0.9923	0.9960	0.9938	0.9515
	*R*_adjusted_^2^	0.9912	0.9965	0.9929	0.9891	0.9910	0.9954	0.9928	0.9434
	*β*	2.23	1.08	1.91	0.92	1.21	0.92	0.84	0.5385
	AIC	70.92	50.81	62.17	64.44	69.44	57.54	69.04	95.752

*R* ^2^: correlation coefficient; *R*_adjusted_^2^: adjusted correlation coefficient using nonlinear regression; AIC: Akaike information criterion; *K*_0_: zero-order release constant; K_1_: first-order release constant; *K*_*h*_: Higuchi rate constant; *K*_*d*_: Hixson–Crowell kinetics constant; *K*_kp_: Korsmeyer release rate constant; *n*: diffusion coefficient; *β*: shape parameter.

## Data Availability

The data used to support the findings of this study are included within the article and are available from the corresponding author upon reasonable request.
